# Trends and disparities in infant mortality from intrauterine hypoxia and birth asphyxia in the United States, 1999–2023

**DOI:** 10.3389/fped.2026.1852806

**Published:** 2026-09-18

**Authors:** Nicole Horio, Keara Kennedy, Kennedy Haase, Olivia Foley, Abubakar Tauseef

**Affiliations:** 1 Creighton University School of Medicine, Omaha, NE, United States; 2 Department of Internal Medicine, Creighton University School of Medicine, Omaha, NE, United States

**Keywords:** birth asphyxia, health disparities, intrauterine hypoxia, mortality trends, neonatal mortality

## Abstract

**Introduction:**

Despite continued advances in prenatal and neonatal care, birth asphyxia and intrauterine hypoxia remain significant causes of preventable mortality in the early neonatal period. Fetal, maternal, and health-system factors influence outcomes immediately after birth. This study aims to analyze trends in infant mortality related to these conditions and examine differences across demographic groups.

**Methods:**

Data on infant mortality related to intrauterine hypoxia or birth asphyxia were obtained from the Centers for Disease Control and Prevention Wide-ranging Online Data for Epidemiologic Research database. Crude mortality rates (CMR) were utilized to calculate annual percent change (APC) and average annual percent change (AAPC) using the Joinpoint Regression Program. Analyses were conducted and stratified by sex, race/ethnicity, urban/rural classification, and United States (US) census region.

**Results:**

A total of 28,500 infant deaths between 1999 and 2023 related to birth asphyxia and intrauterine hypoxia were identified. The overall CMR decreased significantly over this period. Both females and males demonstrated significant decreases in CMR, with AAPCs of -0.90* (95% CI−1.42 to−0.41) and−1.18* (95% CI -1.61 to−0.79), respectively, with male neonates consistently having a higher CMR than females. The CMR significantly decreased in all racial/ethnic groups except NH Asian or Pacific Islander infants. The NH White group showed the greatest decrease in CMR, while the NH Black or African American group consistently had the highest CMR. The Midwest showed the largest decrease in CMR with an AAPC of −1.69* (95% CI−2.54 to−0.85). The CMR also significantly declined in both urban and rural populations, with AAPCs of −1.81* (95% CI−2.42 to−1.26) and −1.50* (95% CI−2.24 to−0.76), respectively.

**Discussion:**

Intrauterine hypoxia and birth asphyxia remain notable causes of neonatal mortality. After early declines, mortality rates have plateaued and modestly increased in recent years. Persistent disparities were observed, with higher mortality among male neonates, non-Hispanic Black neonates, and those in rural and Southern regions. These findings highlight persistent inequities in neonatal care, underscoring the need for targeted, equity-focused interventions.

## 1 Introduction

Infant mortality is a critical indicator of population health, reflecting the quality of pre- and perinatal care as well as broader social and structural determinants shaping neonatal outcomes. Although overall infant mortality due to intrauterine hypoxia and birth asphyxia is relatively low in high-income countries, accounting for less than 0.1% of neonatal deaths, these conditions remain significant causes of preventable mortality (1).

Intrauterine hypoxia reflects impaired gas exchange resulting from compromised placental blood flow (2, 3). Birth asphyxia is clinically defined by failure to initiate or sustain spontaneous breathing at birth. Decreased oxygen delivery leads to fetal hypoxemia, hypercapnia, and metabolic acidosis. This increases the risk for multiorgan injury, hypoxic-ischemic encephalopathy (HIE), and, in severe cases, neonatal death (3). Maternal, fetal, and health-system factors include hypertensive disorders, placental or umbilical complications, pre-term delivery, low birth weight, and limited access to health facilities with a higher level of care (3). Timely recognition and access to obstetric or neonatal intervention are critical to reduce morbidity and mortality (3, 4). Interventions include delivery, neonatal resuscitation, continuous positive airway pressure therapy, neonatal intensive care unit (NICU) services, and trained care providers.

Neonatal mortality is not an exception to the racial inequities seen throughout the health care system in the US. Although Khan et al. found significant overall decline in neonatal mortality due to intrauterine hypoxia and birth asphyxia from 1999 to 2023, Park et al. found persistently higher mortality among non-Hispanic Black infants (5, 6). Similarly, a significantly slower decline in the mortality of black infants highlights the continued discrepancy seen among racial groups, with a 122% higher risk of infant mortality from all causes in black infants than in white infants in 2017 (7). The persistence and exacerbation of these disparities highlight the need for further analysis of demographic and structural trends and how these differences contribute to neonatal mortality.

Despite global research on the epidemiology and clinical determinants of perinatal hypoxia and asphyxia, important gaps remain in understanding long-term demographic and geographic disparities in the US. Existing studies from Brazil and Ethiopia demonstrate differences in outcomes based on population density, facility capability, and prenatal care (3, 8, 9). A seven-year US study found no change in the incidence of HIE, suggesting that persistent factors continue to influence birth outcomes (10). However, demographic and geographic trends and disparities in infant mortality attributable to perinatal hypoxia and asphyxia have not been comprehensively studied. Therefore, this study aimed to evaluate US trends in mortality due to intrauterine hypoxia and birth asphyxia from 1999 to 2023 and to assess variation by sex, race, ethnicity, census region, and urban–rural classification. By examining these trends concurrently, this study seeks to identify populations with persistent mortality disparities and provide population-level evidence to inform targeted strategies to reduce preventable neonatal deaths.

## 2 Methods

Mortality data from 1999 to 2023 was obtained from the Centers for Disease Control and Prevention Wide-ranging Online Data for Epidemiologic Research (CDC WONDER) database (11). Death certificate records of infants <1 year old with intrauterine hypoxia or birth asphyxia-related mortality were obtained from the Multiple Cause-of-Death Public Use Record on the CDC WONDER database. This national registry has been used in prior epidemiologic studies evaluating temporal patterns in mortality from neonatal prematurity (12). Deaths associated with intrauterine hypoxia or birth asphyxia were identified using the International Classification of Diseases, 10th Revision (ICD-10), Clinical Modification codes P20 and P21, respectively. This study is exempt from institutional review board review as the CDC WONDER database provides de-identified, publicly accessible data.

Demographic variables analyzed included sex, race/ethnicity, urban-rural classification, and US Census region. Racial/ethnic categories were based on death certificate designations and included non-Hispanic (NH) White, NH Black or African American, NH Asian or Pacific Islander, and Hispanic or Latino. Urban-rural status followed the National Center for Health Statistics (NCHS) 2013 classification, which categorizes counties as urban (population ≥ 50,000) or rural (population < 50,000) (13). Geographic regions, including Northeast, Midwest, South, and West, were based on US Census Bureau definitions (13).

Crude mortality rates (CMR) related to intrauterine hypoxia and birth asphyxia were calculated by dividing the number of asphyxia-related deaths by the corresponding United States population. Temporal trends in mortality were assessed using the Joinpoint Regression Program (Joinpoint version 5.4.0 available from the National Cancer Institute, Bethesda, Maryland) (14, 15). The Weighted Bayesian Information Criterion (WBIC) was used to determine the ideal model and the optimal number of joinpoints, or trend segments (14, 15). A log-linear model was fitted to each segment between two joinpoints to determine the annual percentage change (APC) (14, 15). Average annual percentage change (AAPC) was calculated from the APCs, and AAPCs were used as a summary of reported mortality for the study period (14, 15). 95% confidence intervals (CIs) were determined for both APC and AAPC using the empirical quantile method, and trends were considered statistically significant if the CI excluded zero (14, 15). Statistically significant results are indicated with asterisks, ‘*’.

## 3 Results

### 3.1 Overall

From 1999 to 2023, there were 28,500 deaths related to neonatal asphyxia and intrauterine hypoxia in the US. The CMR decreased from 44.91 (95% CI 39.31 to 50.52) in 1999 to 25.71 (95% CI 21.57 to 29.86) in 2009, followed by an increase to 32.18 (95% CI 30.34 to 34.02) in 2023 (Figure 1). The overall AAPC in CMR during this entire period was -1.45* (95% CI−1.98 to−0.81) (Supplementary Table 1). The APC from 1999 to 2008 was−5.40* (95% CI−10.85 to−3.10), then increased to 0.99* (95% CI 0.23 to 3.06) from 2008 to 2023 (Supplementary Figure 1).

**Figure 1:**
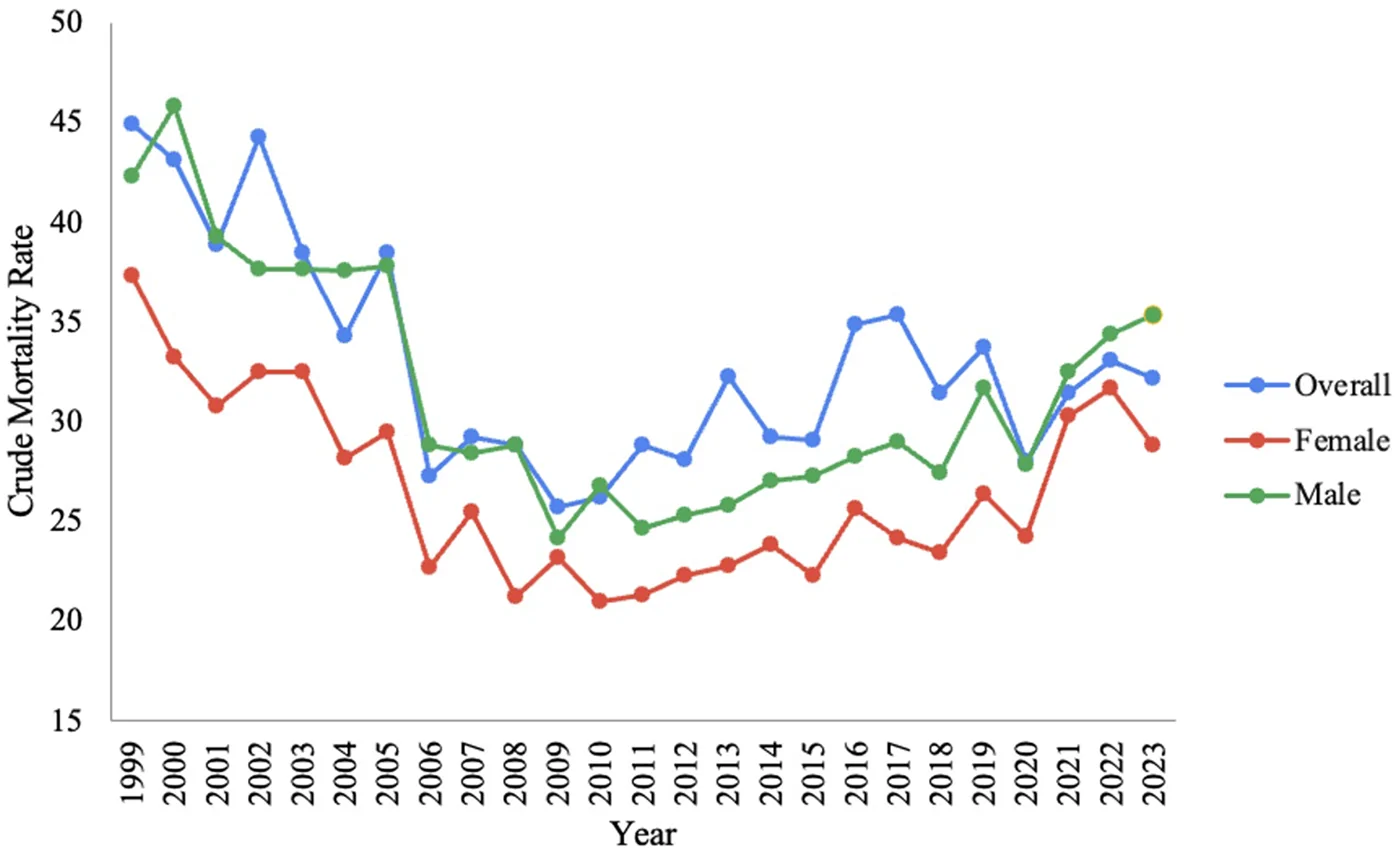
Crude mortality rates related to neonatal asphyxia and intrauterine hypoxia per 100,000 people, overall and stratified by sex, 1999–2023.

### 3.2 Demographic differences

#### 3.2.1 Sex stratified

From 1999 to 2023, neonatal asphyxia and intrauterine hypoxia were contributing factors in 15,814 (55.49%) deaths in males and 12,686 (44.51%) in females in the US, with males having a higher CMR throughout this period.

Among females, the CMR initially steadily decreased from 37.36 (95% CI 34.58 to 40.15) in 1999 to 21.26 (95% CI 21.09 to 25.36) in 2008, before rising again to 28.81 (95% CI 26.32 to 31.30) in 2023 (Figure 1). Despite this increase in recent years, mortality declined overall during the study period (AAPC −0.90*,95% CI−1.42 to−0.41) (Supplementary Table 1). The APC from 1999 to -2010 was 5.01* (95% CI−6.91 to−3.70) and increased to 2.72* (95% CI 1.54 to 4.29) from 2010 to 2023 (Supplementary Figure 1). Among males, the CMR decreased from 42.34 (95% CI 39.45 to 45.24) in 1999 to 24.21 (95% CI 22.08 to 26.35) in 2009, then increased to 35.4 (95% CI 32.70 to 38.10) in 2023. While overall mortality declined among males (AAPC −1.18*, 95% CI−1.61 to−0.79), male neonates consistently had higher mortality rates than females throughout the study period (Supplementary Table 1). The APC was -5.06* (95% CI−6.23 to−4.13) from 1999 to 2011, then increased to 2.85* (95% CI 1.79 to 4.22) from 2011 to 2023 (Supplementary Figure 1).

#### 3.2.2 Race stratified

From 1999 to 2023, deaths related to neonatal asphyxia and intrauterine hypoxia totaled 985 among NH Asian or Pacific Islander infants, 7,205 among NH Black or African American infants, 13,942 among NH White infants, and 5,726 among Hispanic or Latino infants.

NH Black or African American neonates had the highest CMR from 1999 to 2023 compared to NH White and Hispanic or Latino populations, whereas NH Asian or Pacific Islander neonates had the lowest rates across most years (Figure 2). NH Black or African American neonates had a CMR of 66.47 (95% CI 59.76 to 73.17) in 1999, which decreased to 55.40 (95% CI 49.00 to 61.80) in 2023, with an AAPC of −0.83* (95% CI−1.44 to−0.26) (Supplementary Table 2). Temporal APC trends are shown in Supplementary Figure 2.

**Figure 2:**
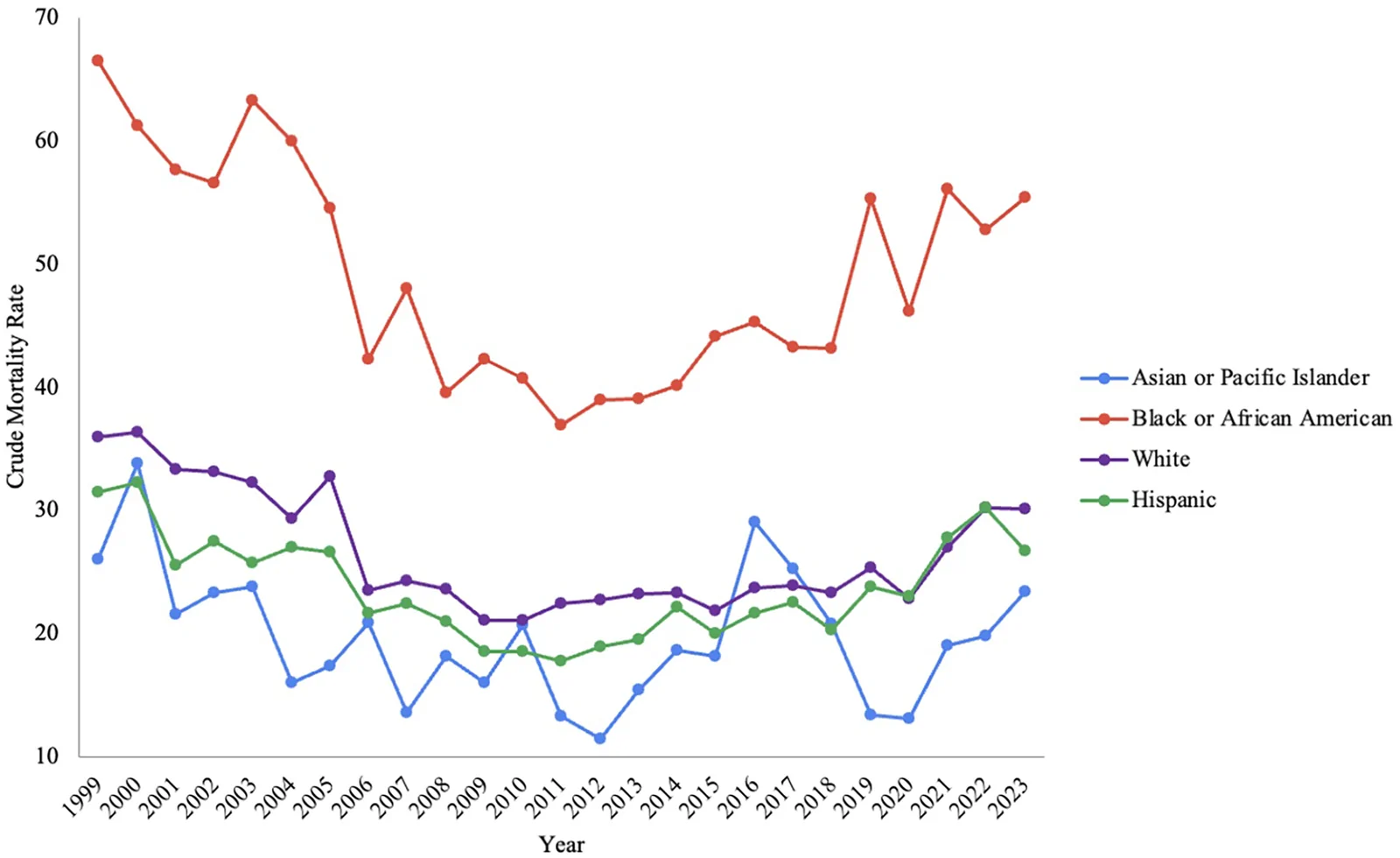
Crude mortality rates related to neonatal asphyxia and intrauterine hypoxia per 100,000 people, stratified by race/ethnicity, 1999–2023.

NH Asian or Pacific Islanders had a CMR of 25.99 (95% CI 18.48 to 35.53) in 1999, which decreased slightly to 23.37 (95% CI 17.17 to 31.08) in 2023, with an AAPC of -0.65 (95% CI -2.29 to 0.71) (Supplementary Table 2).

Among the NH White cohort, the CMR was 35.97 (95% CI 33.51 to 38.43) in 1999 and 30.12 (95% CI 27.52 to 32.73) in 2023, with an AAPC of−1.28* (95% CI−1.76 to−0.86) (Supplementary Table 2). Hispanic or Latino neonates had a CMR of 31.49 (95% CI 27.49 to 35.49) in 1999 and 26.70 (95% CI 23.50 to 29.90) in 2023 with an AAPC of−0.54* (95% CI−1.02 to−0.04) (Supplementary Table 2).

### 3.3 Regional variation

#### 3.3.1 Census region-based differences

Among deaths related to neonatal asphyxia and intrauterine hypoxia, 12,204 occurred in the South, 6,192 in the West, 6,104 in the Midwest, and 4,000 in the Northeast.

All census regions saw a decrease in CMR from 1999 to 2023 (Figure 3). The Midwest had the highest CMR across the study period, decreasing from 45.52 (95% CI 38.14 to 46.89) in 1999 to 31.49 (95% CI 27.46 to 35.53) in 2023, with an AAPC of −1.69* (95% CI−2.54 to−0.85) (Supplementary Table 3). The APC was−3.16 (95% CI−7.22 to 5.35) from 1999 to 2005, decreased to−15.35 (95% CI−19.81 to 8.96) from 2005 to 2008, then increased to 1.91 (95% CI−1.90 to 4.51) from 2008 to 2023 (Supplementary Figure 3). The Northeast consistently had the lowest CMR, with a decline from 37.25 (95% CI 32.59 to 41.90) in 1999 to 24.99 (95% CI 20.94 to 29.04) in 2023, with an AAPC of−1.21* (95% CI−1.88 to−0.59) (Supplementary Table 3).

**Figure 3:**
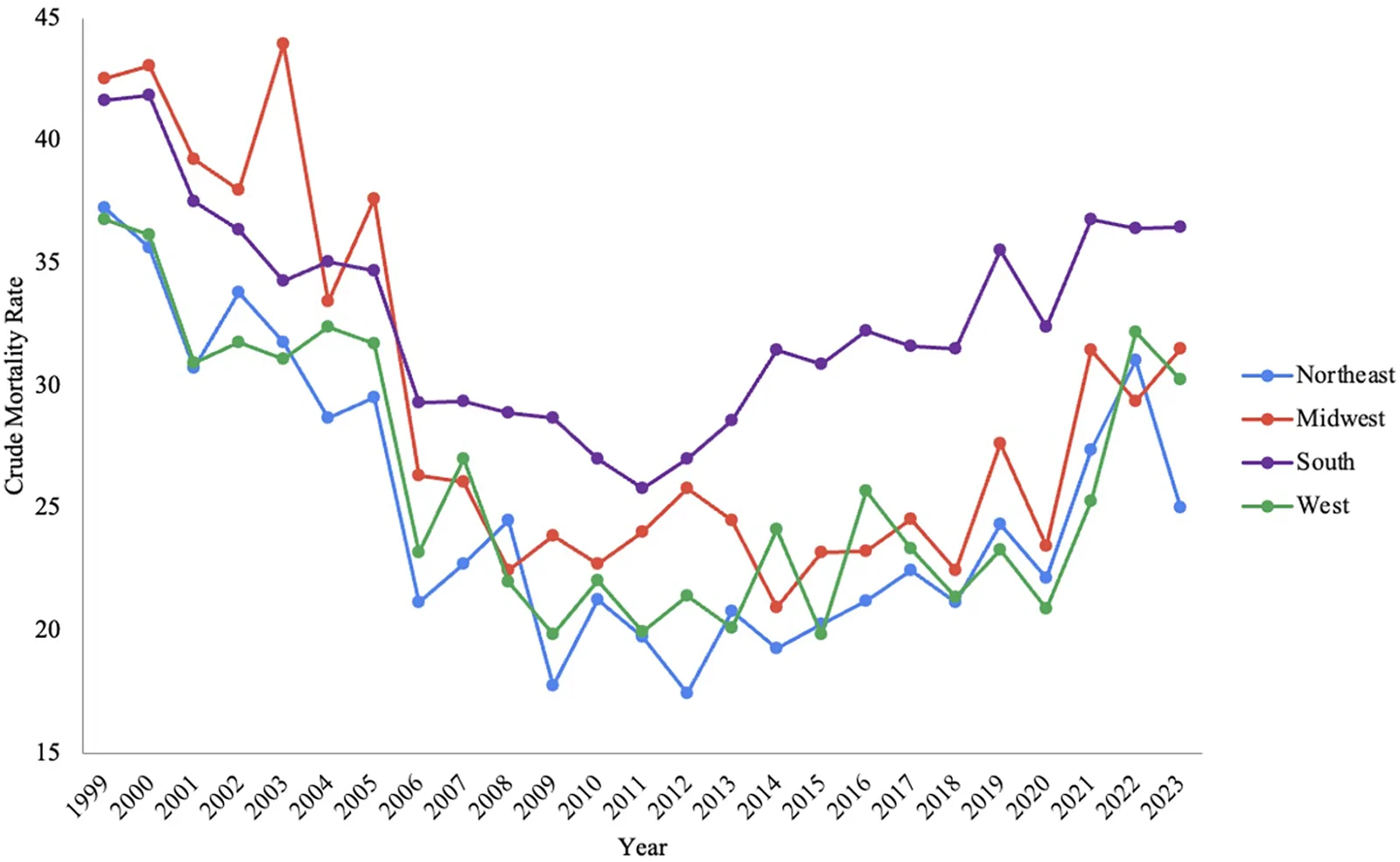
Crude mortality rates related to neonatal asphyxia and intrauterine hypoxia per 100,000 people, stratified by census region, 1999–2023.

The West had a reduction in CMR from 36.79 (95% CI 32.84 to 40.74) in 1999 to 30.25 (95% CI 26.53 to 33.97) in 2023, with an AAPC of−1.23* (95% CI−2.35 to−0.20) (Supplementary Table 3). Lastly, the South had a decrease in CMR from 41.63 (95% CI 38.22 to 45.04) in 1999 to 36.44 (95% CI 33.37 to 39.52) in 2023, with an AAPC of−0.47* (95% CI−0.83 to−0.11) (Supplementary Table 3).

#### 3.3.2 Rural vs. urban

A total of 20,970 neonatal asphyxia and intrauterine hypoxia-related deaths occurred in urban regions, whereas 4,017 occurred in rural regions.

CMR declined in both urban and rural regions from 1999 to 2020; however, urban regions consistently had lower CMRs across the study period (Figure 4). Urban regions had a decrease in CMR from 39.07 (95% CI 36.92 to 41.22) in 1999 to 25.84 (95% CI 24.09 to 27.59) in 2020, with an AAPC of−1.82* (95% CI−2.42 to−1.26) (Supplementary Table 4). The APC was−4.77* (95% CI−6.40 to−3.76) from 1999 to 2011 and increased to 2.28* (95% CI 0.54 to 5.69) from 2011 to 2020 (Supplementary Figure 4). Rural regions had a decline in CMR from 44.91 (95% CI 39.31 to 50.52) to 28.05 (95% CI 23.40 to 32.69), with an AAPC of−1.50* (95% CI−2.24 to−0.76) (Supplementary Table 4). The APC was−5.16* (95% CI−7.86 to−3.55) from 1999 to 2009 and increased to 1.95* (95% CI 0.27 to 4.90) from 2011 to 2020 (Supplementary Figure 4).

**Figure 4:**
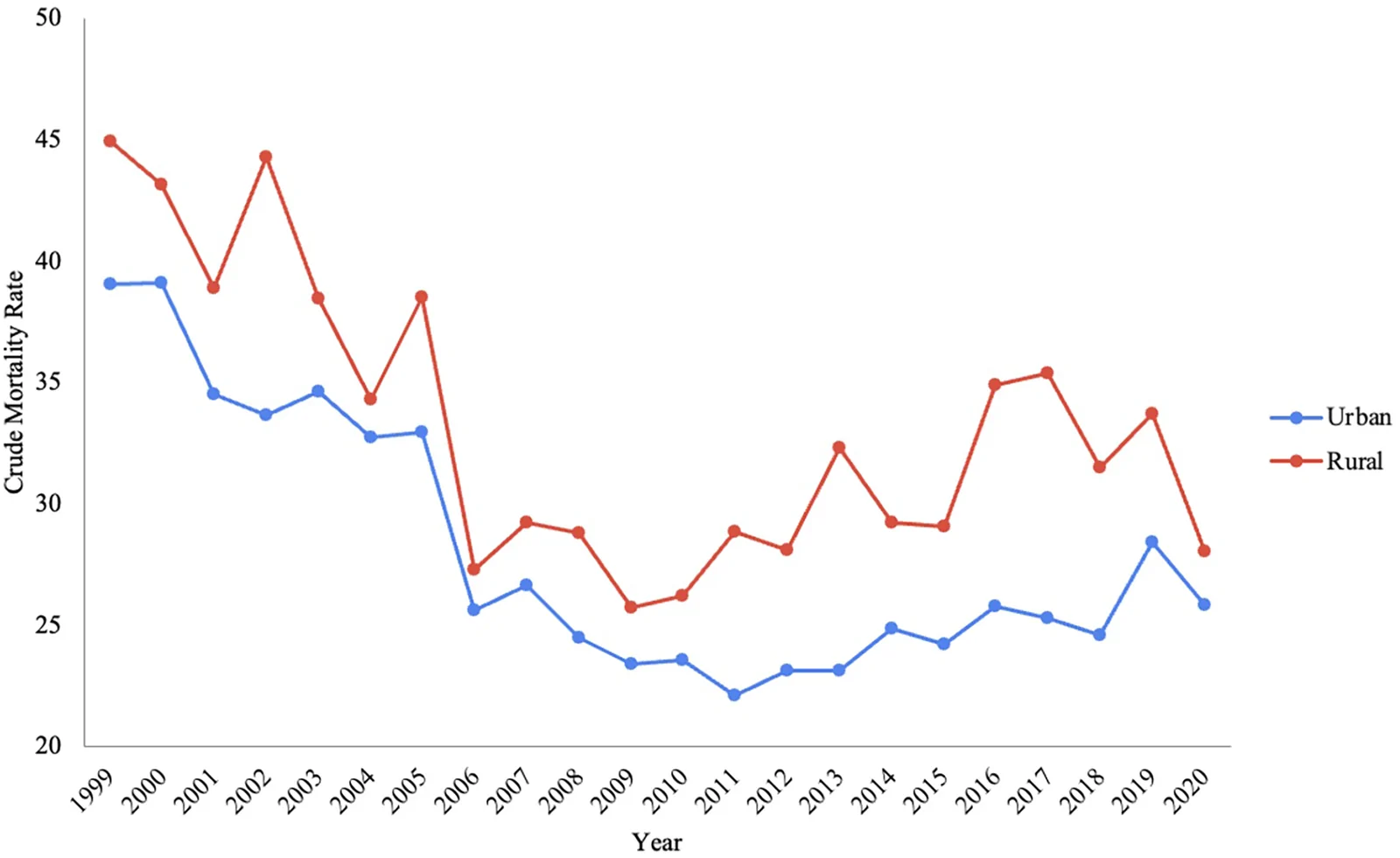
Crude mortality rates related to neonatal asphyxia and intrauterine hypoxia per 100,000 people, stratified by urban vs. rural, 1999–2020.

## 4 Discussion

This study of US mortality due to intrauterine hypoxia and birth asphyxia from 1999 to 2023 identified an important shift in mortality trends. While overall neonatal mortality declined substantially in the early 2000s, rates have plateaued and subsequently increased modestly beginning around 2008. Persistent sex-based disparities were observed, with male neonates consistently experiencing higher mortality rates than females. Additionally, racial and ethnic inequities existed across the study period, with NH Black or African American neonates having higher mortality rates than any other group. Significant geographic variation was identified, with higher mortality rates in the South compared to other census regions and in rural compared to urban areas across the entire study period.

The observed temporal pattern in mortality showed an initial decline (APC of -5.40 from 1999 to 2008) followed by a gradual rise (APC of 0.99 from 2008 to 2023) (Figure 1). Early reductions in mortality may reflect improvements in fetal monitoring, NICU access, and advancements in neonatal resuscitation and treatment of asphyxia. Therapeutic hypothermia is currently the primary intervention shown to reduce mortality among infants with HIE, a potential consequence of intrauterine hypoxia and birth asphyxia. This treatment became first-line in 2010 and has been shown to reduce mortality by 25% (16). During a period of intrauterine hypoxia or birth asphyxia, fetal cardiac output decreases, which in turn decreases cerebral blood flow, leading to impaired delivery of both oxygen and glucose (17). In severe cases, this can lead to permanent necrosis and apoptosis of neurons and ultimately mortality. The use of therapeutic hypothermia reduces cerebral metabolic rate, slowing the process of neuronal death. Additionally, the implementation of neonatal resuscitation training and antenatal interventions has been associated with reductions in neonatal mortality (18). The plateau and overall increase in mortality rates since 2008 suggests the influence of additional factors, including structural barriers and rising maternal comorbidities such as cardiovascular disease (19–21).

Sex-based differences in neonatal mortality were consistent throughout the study period, with male neonates experiencing higher mortality rates than females. This pattern aligns with prior literature demonstrating greater vulnerability among male infants to hypoxic injury. Male sex has been associated with a 1.16-fold increase in severe birth asphyxia (22). Compared to males, females have the added benefit of the protective effects of estrogen at birth. The relative deficiency of this seems to have an effect on brain structure and cellular response that contributes to worse outcomes among male neonates (23).

Racial disparities represented one of the most pronounced findings of this study. NH Black or African American neonates experienced persistently higher mortality rates across the study period, despite overall declines in mortality. Whereas, NH Asian or Pacific Islander neonates had the lowest rates across most years (Figure 2). These findings are supported by inequities found in previous literature in maternal comorbidity burden, as well as disproportionate access to prenatal care and evidence-based neonatal interventions. Previous literature has found that Black infants with HIE are significantly less likely to receive therapeutic hypothermia than White infants, even after adjustment for clinical factors (18). Additionally, structural barriers likely contribute to these disparities. One study found Black women to be more likely to initiate prenatal care late (third trimester) or not at all, compared to White women, at rates of 9.6% and 4.5%, respectively (24). Delays in prenatal care increase neonatal mortality and specifically have been found to increase risk of birth asphyxia by 3.72-fold (25). Lower mortality among Asian and White populations may be associated with lower rates of preterm birth and adverse birth weight, as well as, lower prevalence of maternal risk factors (26). However, outcomes have varied among Asian and Pacific Islander groups, highlighting the heterogeneity within this aggregated racial category (27). Future studies should evaluate interventions aimed at reducing these disparities in access and outcomes.

Regional variation in neonatal mortality due to intrauterine hypoxia and birth asphyxia further highlights the influence of health system factors as well as structural factors on neonatal outcomes. The South consistently faced higher mortality rates compared to other census regions (Figure 3). Previous literature has similarly found higher rates of preterm birth in the South, largely due to racial/ethnic differences. Specifically, the greater proportion of NH Black births are shown to explain 59% of overall regional differences in infant mortality (28). Beyond the South, the discrepancies between census regions may be partially explained by the number of neonatologists and NICU beds, as these factors were found to account for 25.2% of the health service area variance in neonatal mortality, supporting the role of health system capacity in driving regional disparities observed in this study (29). This uneven distribution of NICUs may contribute to the fact that mortality rate in the Midwest declined more rapidly than in the South in the early 2000s. Urban-rural differences further intersect with these regional patterns. While mortality declined in both urban and rural settings, rural regions consistently experienced higher mortality rates than urban regions (Figure 4). These findings may be related to limited access to specialized prenatal obstetric care, delayed neonatal resuscitation, and reduced availability of neonatal intensive care units (30). These findings underscore the need for targeted interventions to address regional and rural inequities in neonatal care. Further investigation should be pursued into strategies to improve access to high-quality prenatal and neonatal services in underserved areas.

### 4.1 Limitations

This study has several limitations. First, this study relied on death certificate data from a publicly available national database, which may be subject to inaccuracies due to incomplete documentation, misclassification, and coding errors. Additionally, this data lacked maternal and neonatal characteristics including gestational age, birth weight, and maternal medical conditions as well as clinical information regarding the timing of resuscitation and receipt of therapeutic hypothermia, limiting causal inference. The study also excluded data from infants identified as belonging to the American Indian or Alaska Native racial/ethnic group when separating the data by race/ethnicity because this designation did not have enough data to be reliable in the analysis. The individuals in this group were still included in all other analyses.

Despite these limitations, this national database study provides a robust assessment of long-term trends and disparities in neonatal mortality secondary to intrauterine hypoxia and birth asphyxia. Future studies integrating population-level mortality data with clinical and health-system information will be critical for identifying modifiable contributors to preventable neonatal deaths and implementing interventions to improve outcomes.

## 5 Conclusion

The aim of this study was to investigate trends in neonatal mortality related to intrauterine hypoxia and birth asphyxia in the US between the years of 1999 and 2023. While there was an observed decrease in CMR over the study period, there were some interesting trends demonstrated in the data. When stratified by sex, male neonates consistently demonstrated a higher CMR than their female counterparts. Stratification by race/ethnicity revealed a significantly increased CMR in NH Black or African American infants than in any other group. Future research may be directed at investigating further into the source of these differences and strategies that might be implemented to eliminate these disparities for the future.

## Data Availability

The original contributions presented in the study are included in the article/Supplementary Material, further inquiries can be directed to the corresponding authors.
