# Supplementary material for: Trends and disparities in infant mortality from intrauterine hypoxia and birth asphyxia in the United States, 1999–2023
Source: Front Pediatr. 2026 Sep 18;14:1852806. doi: 10.3389/fped.2026.1852806 (PMC13630938; doi:10.3389/fped.2026.1852806)
Supplement: Supplementary file 1 [file Datasheet1.pdf]

Supplemental Table I. Mortality Trends Related to Neonatal Asphyxia, Overall and Stratified by Sex, 1999-2023.

	Age-Adjusted Mortality Rate (per 100,000)		
Year	Overall	Female	Male
1999	44.91	37.36	42.34
2000	43.13	33.23	45.82
2001	38.88	30.77	39.32
2002	44.26	32.49	37.70
2003	38.45	32.53	37.67
2004	34.32	28.18	37.57
2005	38.51	29.49	37.80
2006	27.26	22.67	28.85
2007	29.21	25.50	28.43
2008	28.81	21.26	28.82
2009	25.71	23.22	24.21
2010	26.20	20.99	26.76
2011	28.83	21.30	24.66
2012	28.07	22.27	25.29
2013	32.30	22.80	25.83
2014	29.25	23.83	27.01
2015	29.07	22.34	27.27
2016	34.92	25.67	28.27
2017	35.38	24.22	28.98
2018	31.48	23.46	27.43
2019	33.70	26.41	31.73
2020	28.05	24.30	27.88
2021	31.42	30.29	32.50
2022	33.10	31.72	34.42
2023	32.18	28.81	35.40

Number of Joinpoints (Years of Joinpoint)	1 (2008)	1 (2010)	1 (2011)
APC Segment-1 (95% CI)	-5.40* (-10.85 to -3.10)	-5.01* (06.91 to 03.70)	-5.06* (06.23 to 04.13)
APC Segment-2 (95% CI)	0.99* (0.23 to 3.06)	2.72* (1.54 to 4.29)	2.85* (1.79 to 4.22)
Average APC (AAPC) (95% CI)	-1.45* (-1.98 to -0.81)	-0.90* (-1.42 to -0.41)	-1.18* (-1.61 to -0.79)

Supplemental Figure I. Joinpoint model of crude mortality rate related to neonatal asphyxia per 100,000 people overall and stratified by sex, 1999-2023. (* indicates that the APC is statistically significant)

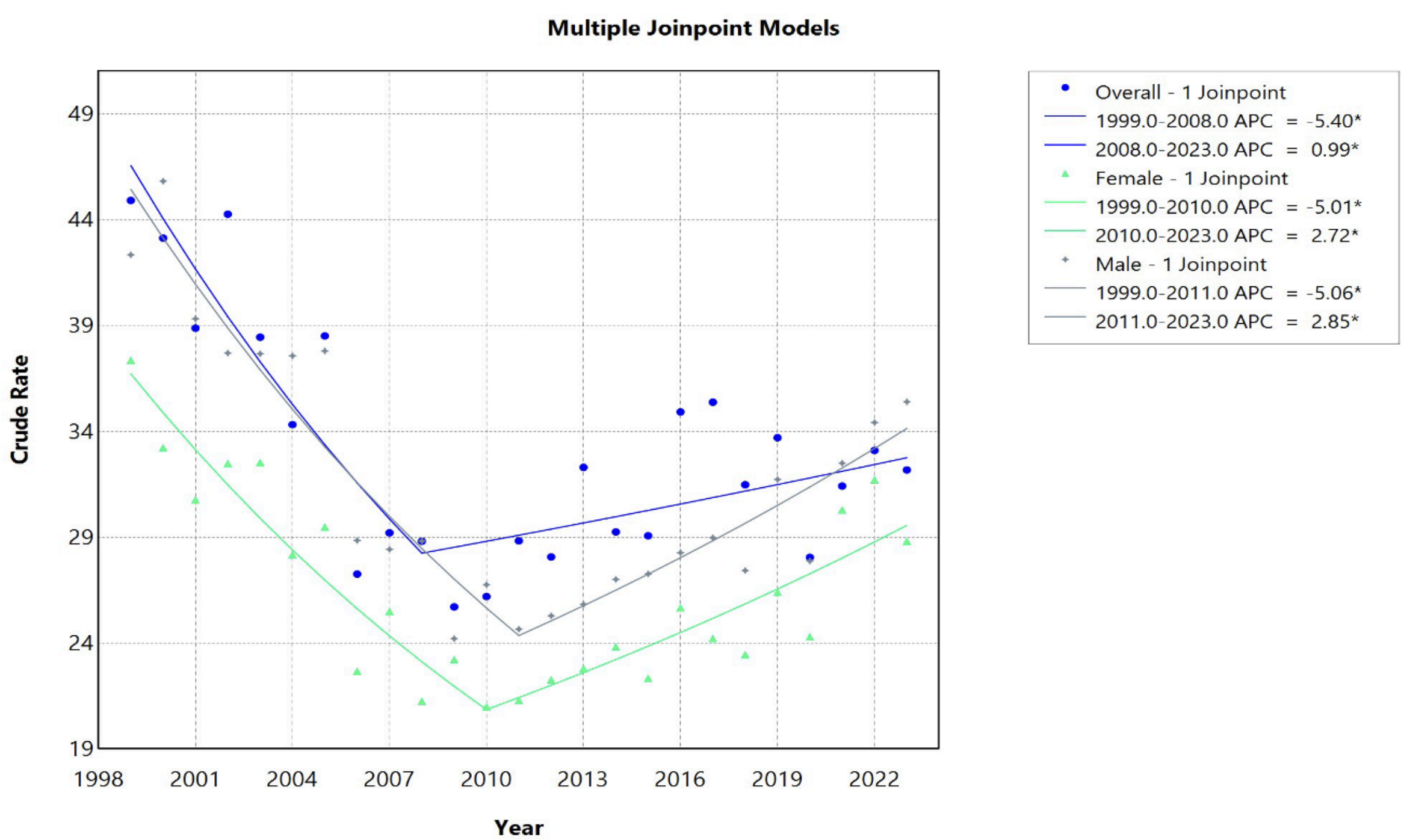

Supplemental Table II. Mortality Trends Related to Neonatal Asphyxia, Stratified by Race and Ethnicity, 1999-2023.

	Age-Adjusted Mortality Rate (per 100,000)			
Year	NH Asian or Pacific Islander	NH Black or African American	NH White	Hispanic/Latino
1999	25.99	66.47	35.97	31.49
2000	33.85	61.19	36.33	32.29
2001	21.61	57.57	33.31	25.58
2002	23.34	56.50	33.14	27.53
2003	23.76	63.20	32.23	25.74
2004	16.06	59.92	29.30	27.03
2005	17.37	54.46	32.72	26.59
2006	20.85	42.22	23.54	21.63
2007	13.61	47.98	24.29	22.47
2008	18.20	39.57	23.63	20.94
2009	15.99	42.26	21.11	18.60
2010	20.65	40.72	21.12	18.57
2011	13.32	36.96	22.47	17.82
2012	11.44	38.94	22.77	18.94
2013	15.41	39.05	23.20	19.51
2014	18.69	40.13	23.28	22.11
2015	18.18	44.07	21.90	20.02
2016	29.04	45.23	23.75	21.65
2017	25.23	43.24	23.86	22.51
2018	20.82	43.09	23.36	20.35
2019	13.44	55.29	25.40	23.79
2020	13.08	46.11	22.80	22.98
2021	19.04	56.04	26.97	27.80
2022	19.81	52.78	30.21	30.26
2023	23.37	55.40	30.12	26.70

Number of Joinpoints (Years of Joinpoint)	3 (2012, 2016, 2020)	1(2011)	1 (2010)	1(2011)
**APC Segment-1 (95% CI)**	-5.67* (-10.24 to -2.59)	-4.76* (-6.74 to -3.44)	-5.21* (-6.69 to -4.14)	-4.67* (-6.19 to -3.48)
**APC Segment-2 (95% CI)**	20.12 (-8.29 to 39.06)	3.27* (1.73 to 5.47)	2.17* (1.13 to 3.50)	3.78 (2.56 to 5.38)
**APC Segment-3 (95% CI)**	-15.73 (-29.29 to 16.37)	–	–	–
**APC Segment-4 (95% CI)**	20.27 (-3.10 to 56.60)	–	–	–
**Average APC (AAPC) (95% CI)**	-0.65 (-2.29 to 0.71)	-0.83* (-1.44 to -0.26)	-1.28* (-1.76 to -0.86)	-0.54* (-1.02 to -0.04)

Supplemental Figure II. Joinpoint model of crude mortality rate related to neonatal asphyxia per 100,000 people overall and stratified by race/ethnicity, 1999-2023. (* indicates that the APC is statistically significant)

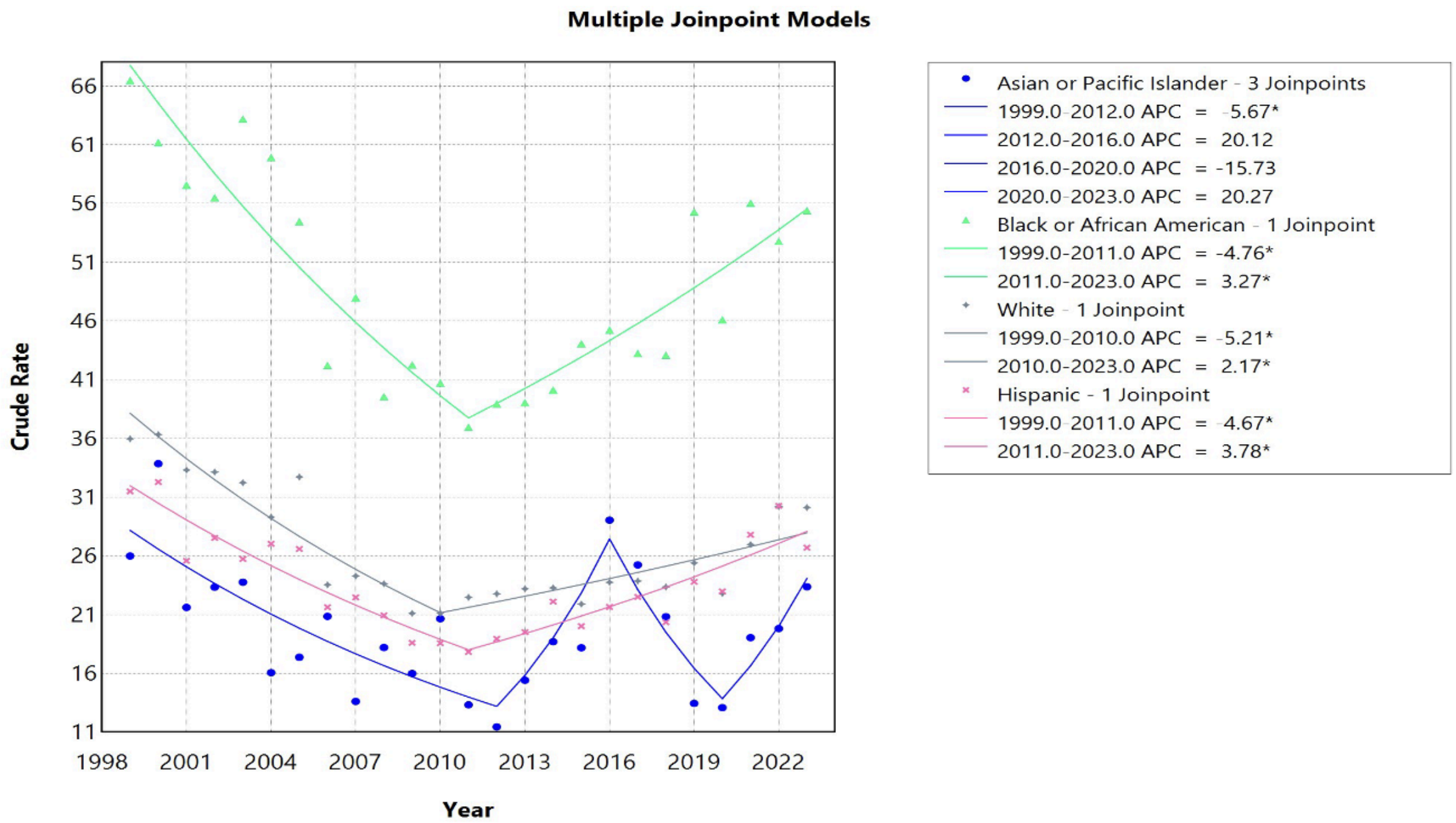

Supplemental Table III. Mortality Trends Related to Neonatal Asphyxia, Stratified by Census Region, 1999-2023.

	Age-Adjusted Mortality Rate (per 100,000)			
Year	Northeast	Midwest	South	West
1999	37.25	42.52	41.63	36.79
2000	35.63	43.04	41.84	36.15
2001	30.72	39.23	37.52	30.89
2002	33.81	37.96	36.35	31.74
2003	31.75	43.95	34.24	31.08
2004	28.68	33.41	35.05	32.40
2005	29.52	37.60	34.70	31.69
2006	21.12	26.30	29.32	23.17
2007	22.69	26.07	29.33	27.01
2008	24.48	22.44	28.89	21.99
2009	17.75	23.88	28.65	19.86
2010	21.24	22.69	26.99	22.04

2011	19.75	24.04	25.79	19.96
2012	17.42	25.77	26.99	21.41
2013	20.77	24.48	28.55	20.11
2014	19.28	20.92	31.42	24.12
2015	20.27	23.19	30.87	19.85
2016	21.22	23.21	32.24	25.67
2017	22.46	24.53	31.58	23.35
2018	21.13	22.46	31.51	21.34
2019	24.35	27.62	35.52	23.28
2020	22.12	23.43	32.39	20.88
2021	27.35	31.46	36.75	25.25
2022	31.02	29.33	36.42	32.16
2023	24.99	31.49	36.44	30.25
**Number of Joinpoints (Years of Joinpoint)**	**1 (2012)**	**2 (2005, 2008)**	**1 (2010)**	**1 (2011)**
**APC Segment-1 (95% CI)**	-5.47* (-7.39 to -4.22)	-3.16 (-7.22 to 5.35)	-4.03* (-5.43 to -3.05)	-5.16* (-11.39 to -2.94)
**APC Segment-2 (95% CI)**	4.08* (2.03 to 7.17)	-15.35 (-19.81 to 8.96)	2.65* (1.83 to 3.74)	2.87* (0.21 to 11.81)
**APC Segment-3 (95% CI)**	–	1.91 (-1.90 to 4.51)	–	–
**Average APC (AAPC) (95% CI)**	-1.21* (-1.88 to -0.59)	-1.69* (-2.54 to -0.85)	-0.47* (-0.83 to -0.12)	-1.23* (-2.35 to -0.20)

Supplemental Figure III. Joinpoint model of crude mortality rate related to neonatal asphyxia per 100,000 people overall and stratified by census region, 1999-2023. (* indicates that the APC is statistically significant)

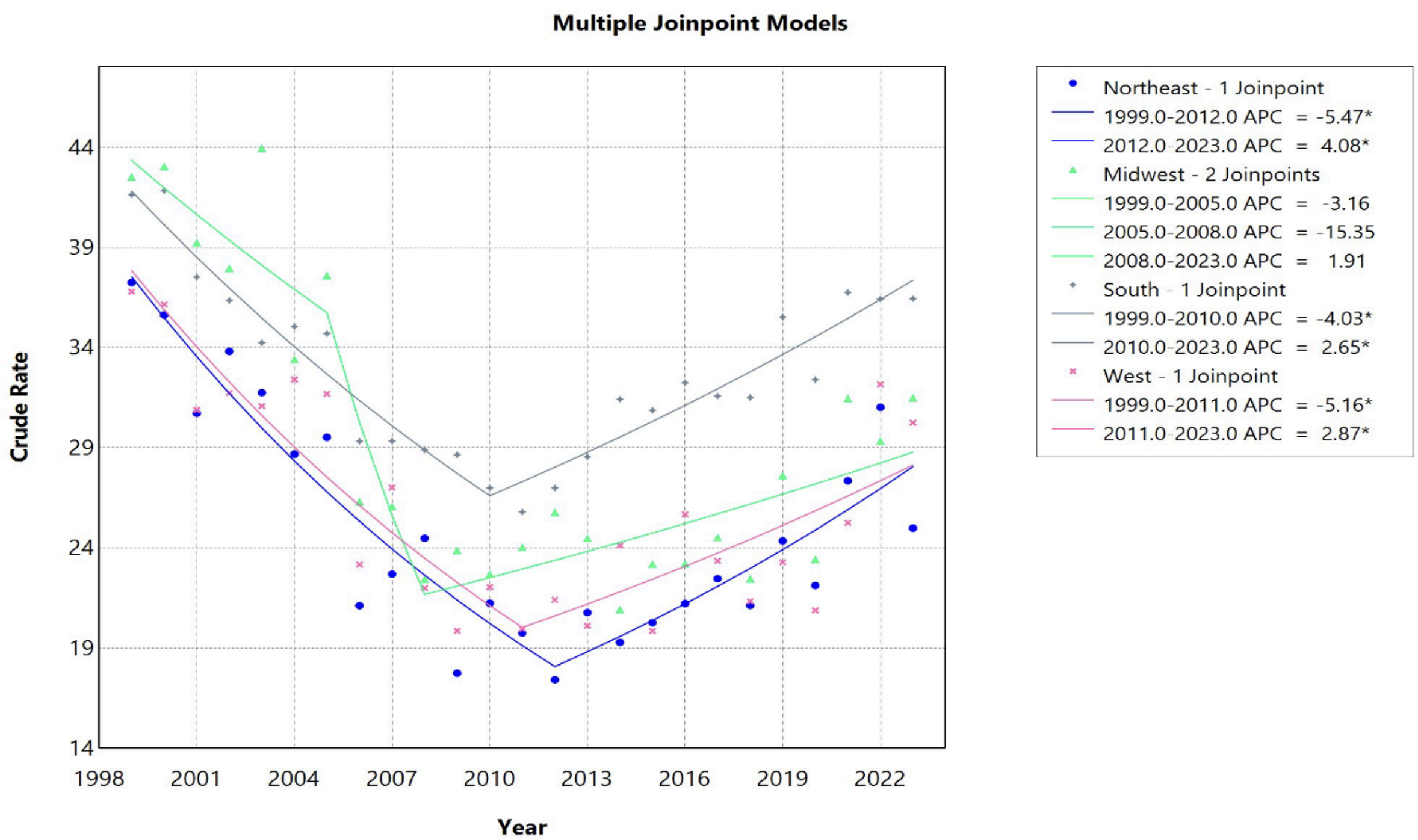

Supplemental Table IV. Mortality Trends Related to Neonatal Asphyxia, Stratified by Urban vs Rural, 1999-2020.

	Age-Adjusted Mortality Rate (per 100,000)	
Year	Urban	Rural
1999	39.07	44.91
2000	39.09	43.13
2001	34.52	38.88
2002	33.66	44.26
2003	34.62	38.45
2004	32.76	34.32
2005	32.94	38.51
2006	25.59	27.26
2007	26.63	29.21
2008	24.49	28.81

2009	23.40	25.71
2010	23.56	26.20
2011	22.10	28.83
2012	23.14	28.07
2013	23.11	32.30
2014	24.86	29.25
2015	24.21	29.07
2016	25.78	34.92
2017	25.31	35.38
2018	24.58	31.48
2019	28.43	33.70
2020	25.84	28.05
**Number of Joinpoints (Years of Joinpoint)**	**1 (2011)**	**1 (2009)**
**APC Segment-1 (95% CI)**	-4.77* (-6.41 to -3.77)	-5.16* (-7.86 to -3.55)
**APC Segment-2 (95% CI)**	2.28 (0.45 to 5.69)	1.95* (0.27 to 4.90)
**Average APC (AAPC) (95% CI)**	-1.81* (-2.42 to -1.26)	-1.50* (-2.24 to -0.76)

Supplemental Figure IV. Joinpoint model of crude mortality rate related to neonatal asphyxia per 100,000 people overall and stratified by urban vs rural, 1999-2020. (* indicates that the APC is statistically significant)

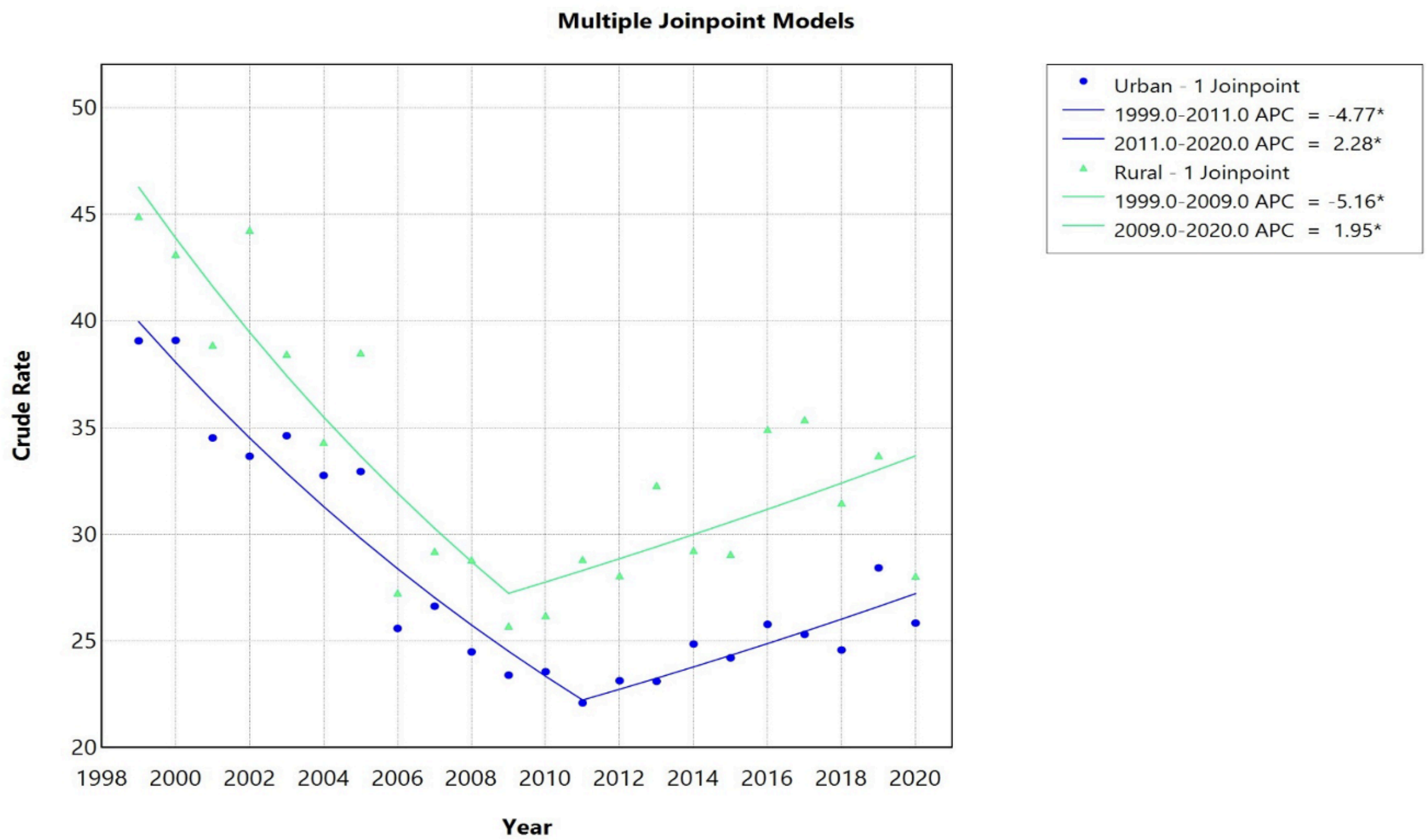

Multiple Joinpoint Models
Crude Rate
Year
50
45
40
35
30
25
20
1998
2000
2002
2004
2006
2008
2010
2012
2014
2016
2018
2020
Urban - 1 Joinpoint
1999.0-2011.0 APC = -4.77*
2011.0-2020.0 APC = 2.28*
Rural - 1 Joinpoint
1999.0-2009.0 APC = -5.16*
2009.0-2020.0 APC = 1.95*
